# Phononic Structure Engineering: the Realization of Einstein Rattling in Calcium Cobaltate for the Suppression of Thermal Conductivity

**DOI:** 10.1038/srep30530

**Published:** 2016-07-26

**Authors:** Ruoming Tian, Gordon J. Kearley, Dehong Yu, Chris D. Ling, Anh Pham, Jan P. Embs, Elvis Shoko, Sean Li

**Affiliations:** 1 School of Materials Science and Engineering, UNSW Australia, Sydney, NSW 2052, Australia; 2 Australian Nuclear Science and Technology Organisation, Lucas Heights, NSW 2232, Australia; 3 School of Chemistry, The University of Sydney, Sydney, NSW 2006, Australia; 4 Paul Scherrer Institute, 5232 Villigen PSI, Switzerland; 5 School of Physics, University of the Witwatersrand, Braamfontein 2000, Johannesburg, South Africa

## Abstract

Phonons in condensed matter materials transmit energy through atomic lattices as coherent vibrational waves. Like electronic and photonic properties, an improved understanding of phononic properties is essential for the development of functional materials, including thermoelectric materials. Recently, an Einstein rattling mode was found in thermoelectric material Na_0.8_CoO_2_, due to the large displacement of Na between the [CoO_2_] layers. In this work, we have realized a different type of rattler in another thermoelectric material Ca_3_Co_4_O_9_ by chemical doping, which possesses the same [CoO_2_] layer as Na_0.8_CoO_2_. It remarkably suppressed the thermal conductivity while enhancing its electrical conductivity. This new type of rattler was investigated by inelastic neutron scattering experiments in conjunction with *ab-initio* molecular dynamics simulations. We found that the large mass of dopant rather than the large displacement is responsible for such rattling in present study, which is fundamentally different from skutterudites, clathrates as well as Na analogue. We have also tentatively studied the phonon band structure of this material by DFT lattice dynamics simulation, showing the relative contribution to phonons in the distinct layers of Ca_3_Co_4_O_9._

Reducing phononic transports without sacrificing mechanical or physical properties is a critical issue for the development of advanced functional materials, including thermoelectric materials. Consequently, there is a massive research effort in the field of thermoelectrics that focuses on finding and developing new semiconductors with low thermal conductivity, but high electrical conductivity and large Seebeck coefficient, these conflicting requirements being a challenge for overall thermoelectric performance^1^,^2^. Chalcogenides, skutterudites, clathrates and pentatellurides have been considered as promising high-performance thermoelectric materials owing to their complex crystal structures^3^,^4^. Incorporating guest atoms that can retain local vibrational dynamics provides so-called rattlers^5^, which can scatter long-range phonons of the host lattice and thus effectively hinder the propagation of heat. These rattlers have become a common approach in the development of thermoelectric materials^6^. For instance, LaFe_3_CoSb_12_ has a value of zT >1 at 700 K, which can be attributed to the reduction of lattice thermal conductivity from La filling as well as the enhancement of carrier concentration^7^. Distinct from phonon rattlers, nanostructures are used to increase the electron density of states at the Fermi level of materials, and to create boundaries or interfaces to scatter phonons. The experimental studies of Bi_2_Te_3_-Sb_2_Te_3_, PbTe–PbSe films and Si nanowires have demonstrated the phonon scattering can reduce lattice thermal conductivity to near *κ*_min_ values (0.2 – 0.5 W m^−1^ K^−1^)^1^. Another example is AgPb_x_SbTe_2+x_, which has a zT of ~2.2 at 800 K in bulk material with nanostructured regions^8^.

In addition to materials engineering with phonon rattlers and nanostructures, phononic crystals (the artificial periodic structures made of two elastic materials) have been used to effectively suppress the thermal conductivity^9^. Periodic changes in density and/or elastic constants in phononic crystals can be manipulated in order to engineer the phononic bandgaps (ranges of the wavelength or frequency within which waves cannot propagate through the structure), filtering out phonons with forbidden frequencies^10^. The ZT value of 2.4 achieved in the Bi_2_Te_3_/Sb_2_Te_3_ superlattice (a typical one-dimensional phononic crystal) was attributed to the significant suppression of thermal conductivity^11^. The assembly of PbTe and Ag_2_Te nanocrystals into a binary 3D superlattice not only reduces the thermal conductivity drastically, but also enhances electrical conductivity by about 2 orders of magnitude over the sum of individual conductivity of single-component PbTe and Ag_2_Te films^12^. Recently, a high-performance centimeter-scale 3D superlattice was intentionally structured with La doped SrTiO_3_ nanocubes coated by 2D electron gas surface made of ultrathin Nb doped SrTiO_3_^13^. Designing such 3D phononic crystals appears to be a promising approach to control the phonon transport while enhancing the electrical conductivity simultaneously. This raises the question of whether a phononic bandgap can be engineered locally by chemical doping, rather than by constructing phononic crystals.

To address this question, we use a layered thermoelectric material [Ca_2_CoO_3_]^RS^[CoO_2_]^H^_δ_ (δ ≈ 1.61, commonly referred to by its approximate composition Ca_3_Co_4_O_9_), as an example to explore the possibility of engineering the phononic structure locally. This compound has a layered structure consisting of disordered rock-salt type [Ca_2_CoO_3_] layers alternating with electrically conductive hexagonal CdI_2_-type [CoO_2_] layers [Fig. 1(a)]. We were prompted to study this material following the observation of Einstein rattling in Na_0.8_CoO_2_, which has similar layered structure. The rattling of Na was found between [CoO_2_] layers^14^. The question then arises whether similar rattling could be provoked in the phononic structure of [Ca_2_CoO_3_] layer in Ca_3_Co_4_O_9_ by substituting elements with masses much larger than the matrix.

Four cobaltate samples were synthesized: pristine CCO (Ca_3_Co_4_O_9+δ_); Bi-doped Bi-CCO (Ca_2.7_Bi_0.3_Co_4_O_9+δ_); Ga-doped Ga-CCO (Ca_3_Co_3.95_Ga_0.05_O_9+δ_); and Bi/Ga co-doped BG-CCO (Ca_2.7_Bi_0.3_Co_3.95_Ga_0.05_O_9+δ_). All the as-prepared materials have similar grain size and relative densities (above 95% of their bulk theoretical density), minimizing the external effects such as porosity and grain size on their intrinsic phononic transport properties. Characterization by laboratory-based X-ray powder diffraction with Cu *Kα* radiation indicated that all these samples were single-phase with the Ca_3_Co_4_O_9_ structure type (Supplementary Figure s1). In order to further study the dopant position in this compound, we carried out synchrotron XRD powder diffraction (with an X-ray wavelength of 0.82565 Å) on the undoped sample and co-doped sample. Rietveld refinement was carried out using *Jana 2006* program. The initial model and refinement procedures have been discussed in our previous structural studies^15^,^16^ on this material. The results indicated that Bi was preferentially doped onto Ca sites and Ga onto Co sites in the rock-salt layer [Ca_2_CoO_3_] (supplementary information).

To study the influence of Bi and Ga substitutions on phonon transports, we have first measured the overall lattice dynamics using inelastic neutron scattering (INS) experiment on CCO and BG-CCO powder samples. Surprisingly, the measured GDOS spectra shown in Fig. 2(a) demonstrate very small modification of GDOS by Bi or Ga doping.

In order to reveal the underlying mechanism, in particular the role of Bi and Ga on the lattice dynamics, we also calculated the phonon density of states using *ab-initio* molecular dynamics (MD) simulation. Distinct from Na_0.8_CoO_2,_ Ca_3_Co_4_O_9_ has more complicated structure, in which a misfit between *b* axis of the two layer types gives rise to the incommensurability δ = *b*_RS_/*b*_H_^17^. For the purposes of simulation, we have approximated the misfit structure to a commensurate model with δ = 8/5 = 1.6 and an overall composition of Ca_3_Co_3.9_O_9.3_. This structure is shown in Fig. 1(a). The starting configuration was then obtained by geometry optimization of the commensurate approximate structure shown in Fig. 1(a) and an equivalent model of BG-CCO with both dopants confined in the [Ca_2_CoO_3_] block (two Bi atoms on Ca sites and one Ga on a Co site), shown in Fig. 1(b). To avoid the uncertainties of empirical force-field methods we have used density function theory (DFT) to determine the forces in MD simulations. The GDOS spectra calculated from the MD atomic trajectories are shown in Fig. 2(b). Although the incommensurate structure of Ca_3_Co_4_O_9_ has been approximated to a commensurate model, the calculated GDOS spectra from MD simulation match the experimental GDOS spectra [Fig. 2(a)] sufficiently well. Similar calculated phonon DOS spectra have been reported by Baran^18^ and Rébola^19^, who carried out the DFT lattice dynamics simulations using a commensurate structure of 5/3 or even 3/2. The general agreement between experiment and simulation suggests that the influence of incommensurability on the lattice dynamics is most likely to be at rather small momentum transfer, which is beyond the current region of study. It also indicates that our MD trajectories can be used to deduce the influence of cation doping on the molecular dynamics of the system.

The partial GDOS spectra of the individual elements in both pristine CCO and co-doped BG-CCO are shown in Fig. 3. As a reference, the spectra of the individual elements in pristine CCO are analyzed. Figure 3(a) demonstrates that Ca in CCO mainly contributes to the energy range 10–40 meV. In contrast, the spectra of Co shown in Fig. 3(b) can be divided into two segments: 10–40 meV with a peak maximum at 28 meV, and a broad feature between 50–80 meV with a maximum at 65 meV, although its phonon density of states in the range 50–80 meV is relatively weak. Similarly, the partial GDOS spectra of O in pristine CCO [Fig. 3(c)] also consists of two parts, 10–50 meV and 50–80 meV with maxima at 35 meV and 65 meV, respectively. The strong GDOS in the energy range 50–80 meV dominates the dynamic behavior of this particular element.

It may also worth looking at the dynamics from individual layers in this material. Since the overall lattice dynamics was almost not changed by doping (Fig. 2), we then investigated the dynamics from two distinct layers by simply considering the undoped material. We have carried out the DFT lattice dynamics simulation with harmonic approximation. The calculated phonon dispersion curves and phonon DOS of individual layers are present in Fig. 4. It is found that the obtained phonon DOS spectra from DFT lattice dynamics calculation [Fig. 4(b,c) is quite similar with the spectra from MD simulation (Fig. 3a–c). It also indicates that there is good consistency between experiment, MD simulation and lattice dynamics calculation.

The dispersion curves (Fig. 4a) show a very large number of branches, and a full analysis of any individual branches is beyond the scope of present work. However, it is encouraging to note that despite the use of approximate commensurate structure, there are only slight negative eigenvalues, which are at the gamma point (cut-off at zero frequency). The dispersion curve shows a gap at around 50 meV, which is also clear in the partial DOS plots illustrated in Fig. 4b,c. Inspection of these plots for Ca and O in the [Ca_2_CoO_3_] layer (Fig. 4b) show rather more intensity below the gap, whilst for O in the [CoO_2_] layer (Fig. 4c), the vast majority is above the gap. To show the relative contribution of Co in each layer, we have normalised the spectra of these two types of Co, subtracted Co_H from Co_RS and plotted the intensity difference in Fig. 4d. It shows that Co in the [Ca_2_CoO_3_] layer has more intensity below 25 meV and less intensity in the range of 25–70 meV.

It appears that although the optical phonons of [Ca_2_CoO_3_] and [CoO_2_] layers are largely independent of each other, there are some residual optical modes involving displacements of elements in both layers. These residual interlayer modes can be identified by detailed analysis of lattice dynamics calculation. However, at present we do not have resources to undertake such analysis. Further experiment and simulation would be carried out in the near future.

Returning to the GDOS spectra, a single peak at 10 meV dominates the GDOS of Bi [Fig. 3(d)], which is fairly isolated from the vibrations of other elements in the compound, this being the hall-mark of an Bi Einstein-oscillator. It is also interesting to note that both experimental and calculated spectra show that the GDOS of BG-CCO is higher than that of pristine CCO in the energy range below 10 meV (Fig. 2). The concentration of Bi is low, but nevertheless, the higher intensity in this energy range for BG-CCO is consistent with the Einstein oscillation of Bi. Beyond this energy range, not only Bi but also Ga, Ca, and Co can contribute to the total phonon density of states.

In general, the vibrational kinetic energy *E* can be expressed as


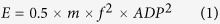


where *m* is the atomic mass, *f* is the frequency, and *ADP* is the atomic displacement parameters. Here, we assign *U* as *ADP*^2^ to simplify the equation. Therefore, the vibrational kinetic energy *E* is proportional to *m* and *U* at a particular frequency. This makes *m *× *U* a key parameter to study the individual contributions of each atom to the total energy *E*. Figure 5 plots the *m *× *U* values against the anisotropic atomic displacement parameters *U* of each atom in the BG-CCO. It demonstrates that Bi has the largest *m *× *U* value among all the elements in BG-CCO, indicating that the Bi rattler absorbs a large portion of kinetic energy in the system. It is also worth noting that the parameters of Bi are comparable to those of the other atoms, implying that the large atomic mass is responsible for the rattling effects, not the large displacements. It is reasonable to consider that, since the mass of Bi is so different from other atoms in the lattice, it oscillates in the time-average mean-field of any other atoms, rather than responding to their dynamics. There could be some weak couplings between Bi and lattice via Ca site at frequencies below 10 meV (the local-mode energy of Bi rattler). But since the interaction is very limited, we only observed very small modification in both experimental INS spectra (Fig. 2a) and MD spectra (Fig. 2b) before and after doping, and the only significant change in the spectra is the additional shoulder around 10 meV due to Bi rattler. This new-type rattling is fundamentally different from skutterudites, clathrates and Na_0.80_CoO_2_ *etc*., in which the displacement parameters of Na rattlers can be an order of magnitude higher than other atoms in the framework, but the mass of Na is rather similar to the other atoms in the lattice^20^.

Although mass-mismatch strategy has been used in alloy-based thermoelectric materials to hinder phonon propagation^21^,^22^, our system is too complex to be treated in the theories. In order to further study such mass-mismatch effect in our system, we carried out the *ab-initio* MD simulation with a substitute atom having the same electronic structure, atomic size and chemical environment of Bi but an atomic mass of 122 (equivalent to Sb). The calculated GDOS for Bi and this substitute atom are shown in Fig. 6. It shows that the characteristic peak of GDOS for the nominal atom, which is near half of the atomic mass of Bi, becomes much broader and the corresponding frequency is increased by ~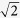. This agrees well with the expectation from Equation (1) that the vibration frequency *f* should increase by 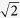
when the dopant losses its half mass, indicating that the change of vibrational kinetic energy caused by atomic displacements is very limited. It also suggests that the oscillation mass could be simplified to the dopant mass in the current system. In addition, the light substitute-atom shifts its vibrational frequencies to the range in which other elements vibrate, resulting in a more effective vibrational mixing. Most importantly, it further demonstrates that it is the atomic mass that plays the key role in determining the phonon transport in this particular layered compound.

To elucidate the influence of temperature on the Einstein oscillation, we further experimentally investigated the dynamical behavior in the energy range 0–20 meV at three different temperatures, 400 K, 800 K and 1000 K [Fig. 7(a–c)] at PSI. The result demonstrates that the GDOS of BG-CCO at E < 10 meV is higher than that of its pristine counterpart at all the measurement temperatures, which is in good agreement with the simulated results. In order to provide a direct comparison, we also plotted the ratio of BG-CCO experimental GDOS spectra to the pristine CCO experimental GDOS spectra over the full range of energies at the measured temperatures in Fig. 8. The difference between the doping engineered BG-CCO and the pristine CCO spectra is distinct at energies below 10 meV, which is mainly contributed by the Einstein-like rattling of Bi. It is also noteworthy that such a local vibration mode persists at all temperatures, suggesting that the Bi is a good rattler, which is fairly independent of host lattice.

To further understand the consequence of the observed phononic behaviors in the as-prepared materials, the thermal conductivities as a function of temperature for the samples of CCO, Ga-CCO, Bi-CCO and BG-CCO were measured and are plotted in Fig. 9(a). This shows that both Bi and Ga doping can reduce the thermal conductivity over the measurement temperature range. The reduction was found to be more significant with the presence of Bi. In particular, the BG-CCO presents the lowest thermal conductivity, which is ~40% lower than that of the pristine CCO. We used the Wiedemann-Franz law to separate the individual thermal conductivities contributed to by hole carriers (κ_h_) and phonons (κ_ph_). The results are plotted in Fig. 9(b), in which it can be clearly seen that the reduction of total thermal conductivity is mainly due to the suppression of phonon transport through Bi rattler. On the other hand, the mass of Ga is similar to both Ca and Co, and in this case we do not observe a single sharp Einstein mode in the partial phonon DOS of Ga (Fig. 3d). Thus the reduction of thermal conductivity from Ga doping is less significant. In addition, we have conducted the measurement of electrical resistivity on both undoped and doped materials (Supplementary Table s1), which shows that the electrical resistivity was actually decreased by doping over the measured temperature range. This suggests that not only thermal conductivity was reduced by doping, but there is also a slight enhancement in the electrical conductivity, both being favorable for thermoelectric applications. A full understanding of electronic structure by chemical doping should pave the way to considerable improvement in the thermoelectric performance.

In summary, we have realized a new type of rattler in thermoelectric material Ca_3_Co_4_O_9_ by substituting Bi for Ca site in the central block between [CoO_2_] layers. Inelastic neutron scattering experiment shows that doping has very limited influence on the overall lattice dynamics. However, MD simulations identify the Bi rattler at 10 meV, which is local and fairly independent of host lattice. It remarkably reduced the thermal conductivity while slightly enhancing the electrical conductivity. More importantly, the rattling is found to be resulted from the big contrast of atomic mass between dopant and other elements in the lattice rather than the large displacement of dopant. This is essentially and fundamentally different from skutterudites, clathrates and Na_x_CoO_2_, which may bring new insights into the design of better thermoelectric materials.

## Methods

### Sample preparation

Stoichiometric ratios of CaCO_3_ (99.0%, Sigma Aldrich), Bi_2_O_3_ (99.5%, Sigma Aldrich), and Co_3_O_4_ (99.7%, Alfa Aesar) powders were mixed and ground using ball-milling in zirconia media with ethanol for 12 h. The mixed powders were then dried and calcined twice at 1173 K for 20 h in air with intermediate regrinding. The products were ball-milled again and placed into a 20 mm graphite mould. Spark plasma sintering was carried out in a Dr Sinter SPS-825 (Syntex, Inc., Japan) system under vacuum. Prior to sintering, a moderate pressure of ~10 MPa was applied to the mould to ensure a closed electrical loop for the current to pass through. The sample was then heated to 1073 K under a uniaxial pressure of 50 MPa and held for 5 min. The sintered pellets were annealed at 1173 K in air for 20 h and cooled to room temperature at a rate of 5 K/min to remove the graphite foil on the surface and re-oxidise them to the same state.

### Measurement of thermal properties

Thermal diffusivity, heat capacity and thermal expansion were measured by a laser flash system (NETZSCH LFA-427), differential scanning calorimeter (NETZSCH DSC-404C) and dilatometer (NETZSCH DIL-402C), respectively. Thermal conductivity was evaluated from κ = *DρC*_*p*_ where *D* is thermal diffusivity, *ρ* is density and *C*_*p*_ is heat capacity.

### Inelastic neutron scattering experiment

The inelastic neutron scattering (INS) experiments were performed using the time-of-flight neutron spectrometer FOCUS with an incident neutron wavelength of λ = 4 Å at the Paul Scherrer Institute in Switzerland. Energy transfer was measured up to 100 meV. Data for both pristine and co-doped samples were collected at 400, 800 and 1000 K. All the measured spectra were normalized to a standard vanadium sample and corrected for the scattering from the empty scan. The obtained data were converted into the scattering function *S* (*q*, *w*) using the DAVE package^23^ and the generalized density of states (GDOS) was derived subsequently^24^.

### Molecular dynamics simulation

The plane wave density functional theory (DFT) code (VASP)^25^ was used for all MD simulations. The calculation consists of two parts: geometry optimization to determine the minimum-energy starting configuration, and then MD simulations to determine the dynamics at a given temperature. Production simulations in the microcanonical ensemble at 298 K, were developed from the minimum-energy configuration in the following way: (i) 4 *ps* of equilibration in the isokinetic ensemble (i.e., velocity scaling) with the temperature fixed to T; (ii) 4 *ps* of equilibration in the microcanonical ensemble; and (iii) at least 8 *ps* of production in the microcanonical ensemble with the thermodynamic temperature fluctuating about T. In all VASP calculations, we adopted the projector augmented wave (PAW) potential^26^ and the Perdew-Burke-Ernzerhof (PBE) exchange-correlation function^27^. For all MD simulations, the energy cutoff was reduced to 300 eV and a time step of 1 *fs* was used.

**Figure 2:**
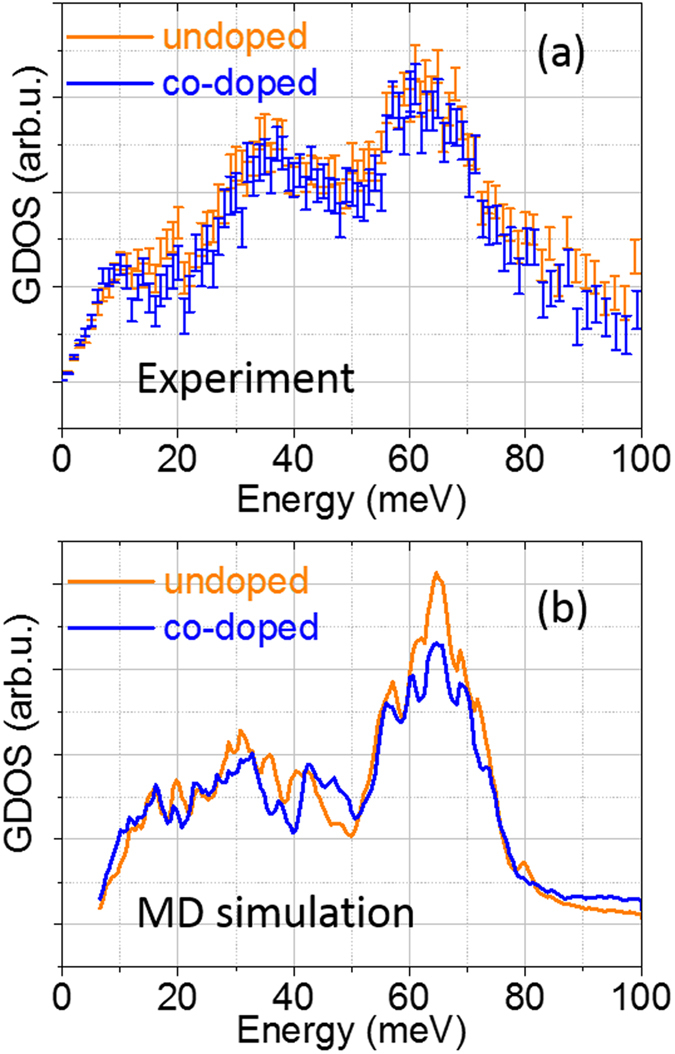
Measured INS spectra (as GDOS) for undoped and co-doped samples at 400 K, and (**b**) the calculated GDOS spectra for the corresponding samples from *ab-initio* MD simulations at 298 K. The elastic Q range is from 0.1 to 2.7 Å^−1^ and inelastic one is from 0.1 to 12.5 Å^−1^.

The dynamical structure factor, *S*(*q*, *w*) was calculated from the atomic trajectories of the MD simulations using the code NMoldyn^28^. Firstly, the incoherent intermediate scattering function, *F*(*q*, *t*) was calculated on a regular grid in both momentum transfer *q* and time *t*. Then *F*(*q*, *t*) was Fourier-transformed to obtain the scattering function, *S*(*q*, *w*). In-house software was used to take the appropriate cut was taken through this surface to coincide with the experimental INS data, and this was then convoluted with the instrumental resolution function to obtain the calculated INS spectrum, which is compared with the observed spectrum in Fig. 2.

### Lattice dynamics simulations

**Figure 4:**
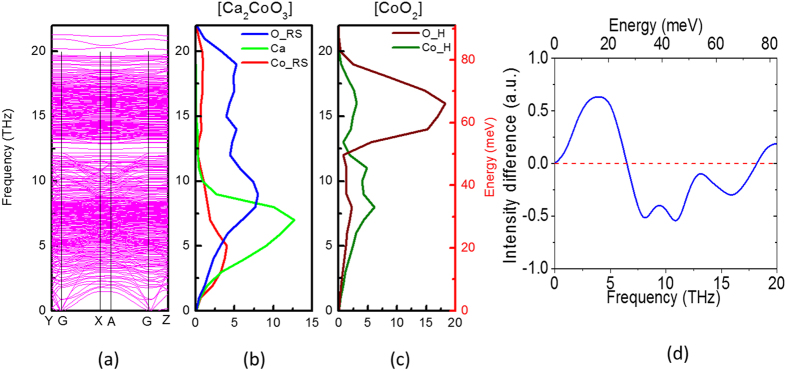
(**a**) Calculated phonon dispersion curves, (**b**) partial phonon DOS spectra in the [Ca_2_CoO_3_] layer, (**c**) partial phonon DOS spectra in [CoO_2_] layer from DFT lattice dynamics simulation, (**d**) the intensity difference of two types of Co by normalizing the individual spectrum and subtracting Co_H from Co_RS.

The DFT + U were calculated using the projected augmented wave (PAW)^29^ method with the Perdew- Burke and-Ernzerhof (PBE)^27^ functional and the Dudarev’s scheme^30^ within the VASP code. A value of U = 4 eV was included in the Co’s 3d to account for the strongly corrected effect in Ca_3_Co_4_O_9_ as described in the previous study^31^. To calculate the phonon dispersion and the contribution of the individual ions in the phonon density of states (PDOS), a supercell consisting of 108 atoms with a mismatch ratio of ~8/5 between the CoO_2_ layer and the rocksalt layer, with the experimental lattice constants obtained from the synchrotron diffraction study. The internal coordinates of the individual ions were first relaxed till the forces less than 0.004 eV/atom and an energy convergence of 10^−7^. A cut-off energy of 550 eV and a dense Monkhorst-Pack^32^ k-point set of 4 × 2 × 2. The set of forces was calculated using the Finite Displacement Method on the phonon band structure (Fig. 4a) and partial DOS (Fig. 4b,c) were then extracted through the PHONOPY^33^ software from the first principle calculation. The partial DOS was calculated using a Γ-centred k-point mesh with 960 kpoints.

## Additional Information

**How to cite this article**: Tian, R. *et al*. Phononic Structure Engineering: the Realization of Einstein Rattling in Calcium Cobaltate for the Suppression of Thermal Conductivity. *Sci. Rep.* **6**, 30530; doi: 10.1038/srep30530 (2016).

## Supplementary Material

Supplementary Information

## Figures and Tables

**Figure 1:**
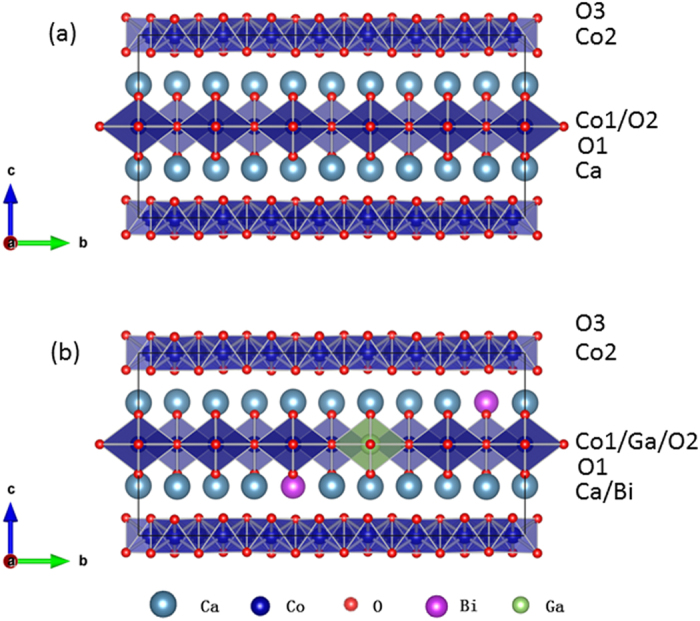
The commensurate approximate (*q* = 1.6) supercells of (**a**) undoped and (**b**) the Bi/Ga co-doped CCO. The former consists of 20 Ca (grey), 26 Co (blue) and 62 O (red) atoms, while the latter consists of 18 Ca, 25 Co and 62 O atoms with 2 Bi (purple) on Ca sites and 1 Ga (green) on a Co site in the [Ca_2_CoO_3_] layer. The primitive cell parameters are *a* = 4.835, *b* = 22.72, *c* = 10.85 Å and *β* = 98.12°.

**Figure 3:**
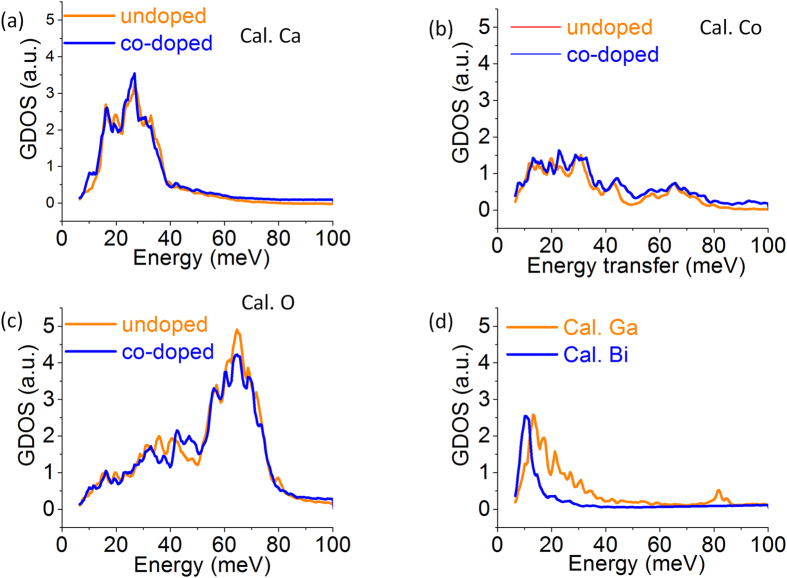
Calculated partial GDOS spectra of (**a**) Ca, (**b**) Co, (**c**) O and (**d**) Ga and Bi from the MD simulations.

**Figure 5:**
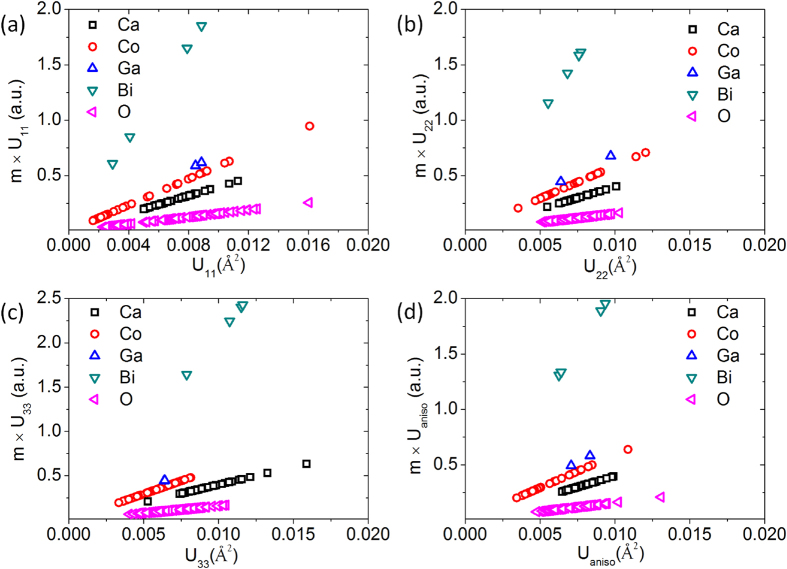
Plot of *m *× *U* as a function of anisotropic atomic displacement parameter *U*.

**Figure 6:**
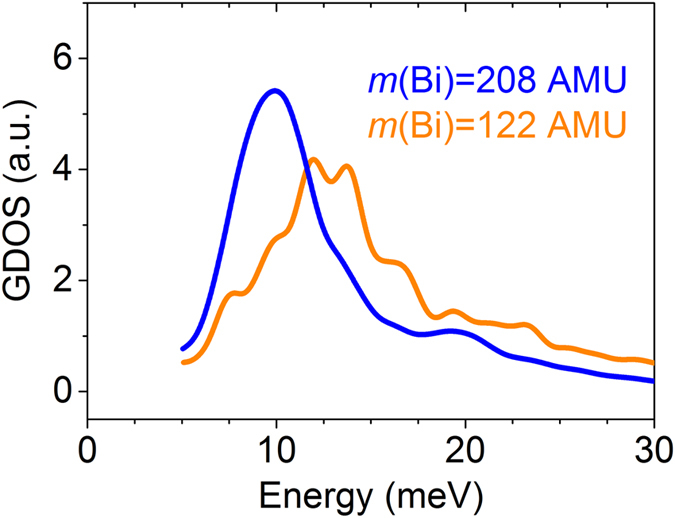
Calculated partial density of states for Bi rattlers with mass of 208 amu (blue) and artificial mass of 122 amu (orange), equivalent to Sb.

**Figure 7:**
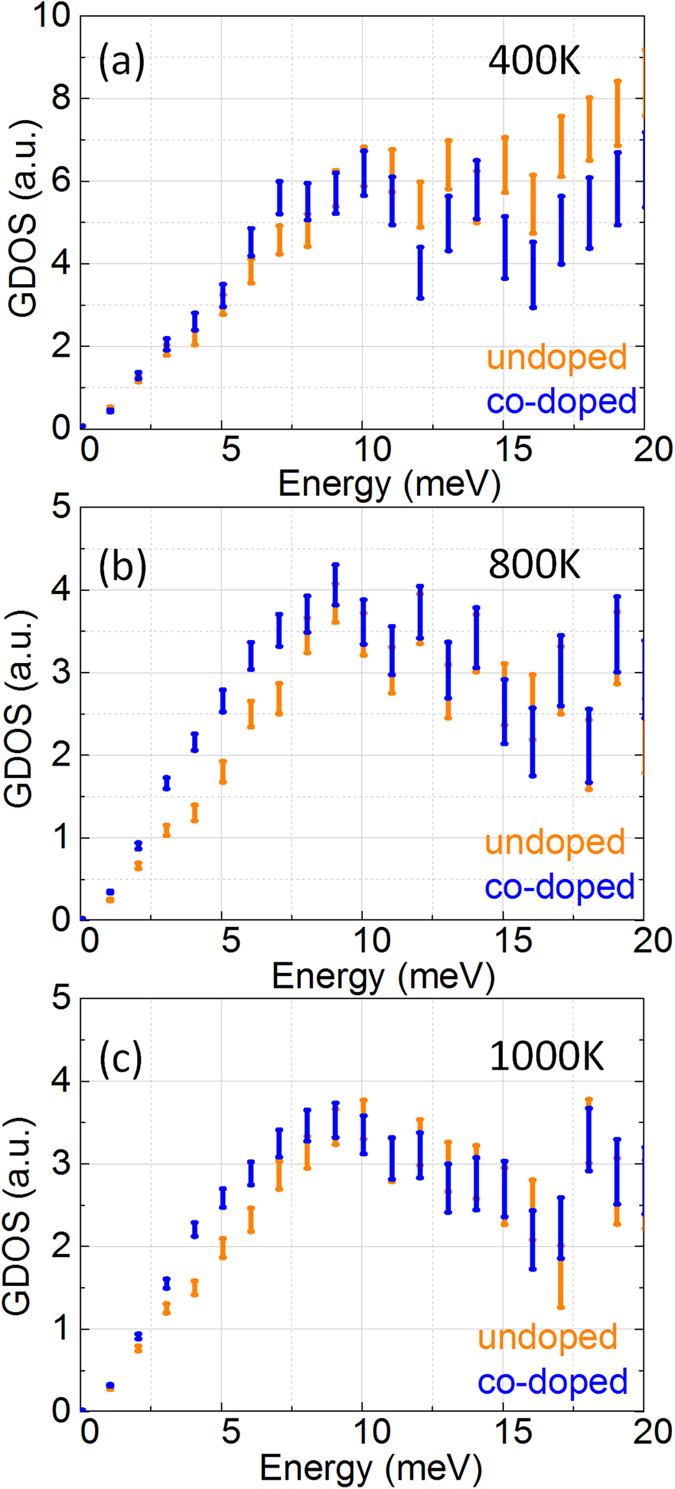
GDOS spectra measured by INS experiment at (**a**) 400 K, (**b**) 800 K and (**c**) 1000 K in the range 0–20 meV.

**Figure 8:**
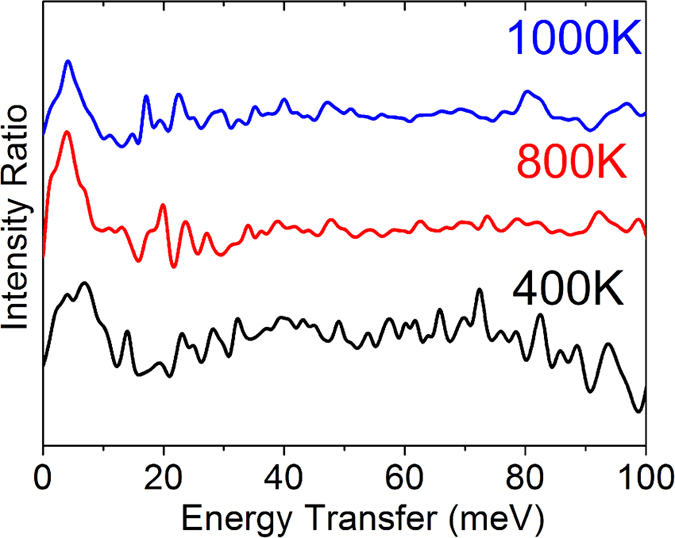
The intensity ratio of the observed co-doped spectrum over undoped spectrum at three different temperatures 400 K, 800 K and 1000 K. The higher relative intensity of the co-doped spectrum around 10 meV is a clear indication of extra dynamics of the co-doped sample in this region, which is consistent with the analysis of MD simulation.

**Figure 9:**
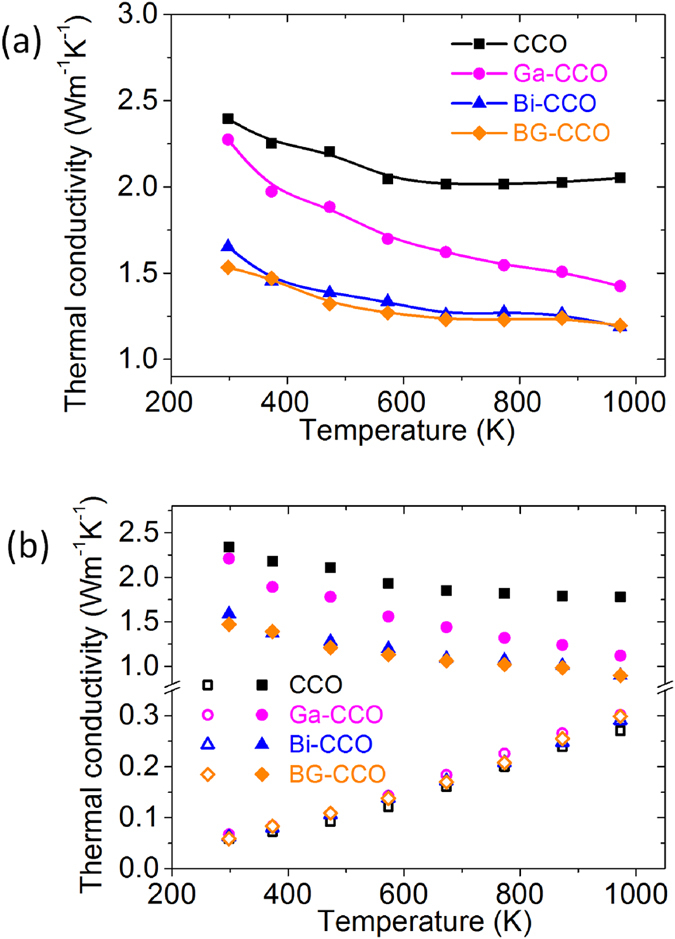
(**a**) Temperature dependence of thermal conductivity for the four representative calcium cobaltate samples and (**b**) temperature dependence of phonon thermal conductivity κ_ph_ (solid symbols) and electron thermal conductivity κ_h_ (hollow symbols) of the investigated samples.

## References

[b1] Snyder G. J. & Toberer E. S. Complex thermoelectric materials. Nature Mater. 7, 105–114 (2008). 18219332 10.1038/nmat2090

[b2] Daniel P. In CRC Handbook of Thermoelectrics (CRC Press, 1995).

[b3] Koza M. M. . Breakdown of phonon glass paradigm in La- and Ce-filled Fe_4_Sb_12_ skutterudites. Nature Mater. 7, 805–810 (2008). 18758457 10.1038/nmat2260

[b4] Christensen M. . Avoided crossing of rattler modes in thermoelectric materials. Nature Mater. 7, 811–815 (2008). 18758454 10.1038/nmat2273

[b5] Keppens V. . Localized vibrational modes in metallic solids. Nature 395, 876–878 (1998).

[b6] Tritt T. M. Holey and unholey semiconductors. Science 283, 804–805 (1999).

[b7] Sales B., Mandrus D. & Williams R. K. Filled skutterudite antimonides: a new class of thermoelectric materials. Science 272, 1325–1328 (1996). 8662465 10.1126/science.272.5266.1325

[b8] Hsu K. F. . Cubic AgPb_m_SbTe_2+m_: bulk thermoelectric materials with high figure of merit. Science 303, 818–821 (2004). 14764873 10.1126/science.1092963

[b9] Yang L., Yang N. & Li B. Reduction of thermal conductivity by nanoscale 3D phononic crystal. Sci. Rep. 3 (2013). 10.1038/srep01143 PMC3560355 23378898

[b10] Gorishnyy T., Maldovan M., Ullal C. & Thomas E. Sound ideas. Phys. World 18, 24–29 (2005). 10.1103/PhysRevLett.94.115501 15903869

[b11] Venkatasubramanian R., Siivola E., Colpitts T. & O’quinn B. Thin-film thermoelectric devices with high room-temperature figures of merit. Nature 413, 597–602 (2001). 11595940 10.1038/35098012

[b12] Urban J. J., Talapin D. V., Shevchenko E. V., Kagan C. R. & Murray C. B. Synergism in binary nanocrystal superlattices leads to enhanced p-type conductivity in self-assembled PbTe/Ag_2_Te thin films. Nature Mater. 6, 115–121 (2007). 17237786 10.1038/nmat1826

[b13] Koumoto K. . Thermoelectric ceramics for energy harvesting. J.Am.Ceram.Soc 96, 1–23 (2013).

[b14] Voneshen D. . Suppression of thermal conductivity by rattling modes in thermoelectric sodium cobaltate. Nature Mater. 12, 1028–1032 (2013). 23975057 10.1038/nmat3739

[b15] Ling C. D., Aivazian K., Schmid S. & Jensen P. Structural investigation of oxygen non-stoichiometry and cation doping in misfit-layered thermoelectric [Ca_2_CoO_3−x_][CoO_2_]_δ_, δ~1.61. J. Solid State Chem. 180, 1446–1455 (2007).

[b16] Tian R. . Ga Substitution and Oxygen Diffusion Kinetics in Ca_3_Co_4_O_9+δ_-Based Thermoelectric Oxides. J. Phys. Chemi. C 117, 13382–13387 (2013).

[b17] Lambert S., Leligny H. & Grebille D. Three Forms of the Misfit Layered Cobaltite [Ca_2_CoO_3_] [CoO_2_]_1.62_·A 4D Structural Investigation. J. Solid State Chem. 160, 322–331 (2001).

[b18] Baran J. D. . Tuning thermoelectric properties of misfit layered cobaltites by chemically induced strain. J. Phys. Chem. C 119, 21818–21827 (2015).

[b19] Rébola A., Klie R. F., Zapol P. & Öğüt S. Phonon and thermal transport properties of the misfit-layered oxide thermoelectric Ca_3_Co_4_O_9_ from first principles. Appl. Phys. Lett. 104, 251910 (2014).

[b20] Goldsmid H. J. Introduction to thermoelectricity. Vol. 121 (Springer, 2009).

[b21] Delaire O. . Heavy-impurity resonance, hybridization, and phonon spectral functions in Fe _1− x_ M _x_ Si (M = Ir, Os). Phys. Rev. B 91, 094307 (2015).

[b22] Ziman J. M. & Armstrong H. Principles of the Theory of Solids. American Journal of Physics 33, 349–350 (1965).

[b23] Azuah R. T. . DAVE: a comprehensive software suite for the reduction, visualization, and analysis of low energy neutron spectroscopic data. Journal of Research of the National Institute of Standards and Technology 114, 341–358 (2009). 10.6028/jres.114.025 PMC4646530 27504233

[b24] Furrer A., Mesot J. & Strässle T. Neutron scattering in condensed matter physics. Vol. 4 (World Scientific Pub Co Inc, 2009).

[b25] Kresse G. & Furthmüller J. Efficient iterative schemes for ab initio total-energy calculations using a plane-wave basis set. Phys. Rev. B 54, 11169 (1996). 10.1103/physrevb.54.11169 9984901

[b26] Blöchl P. E. Projector augmented-wave method. Phys. Rev. B 50, 17953 (1994). 10.1103/physrevb.50.17953 9976227

[b27] Perdew J. P., Burke K. & Ernzerhof M. Generalized gradient approximation made simple. Phys. Rev. Lett. 77, 3865 (1996). 10062328 10.1103/PhysRevLett.77.3865

[b28] Kresse G. & Hafner J. Ab initio molecular dynamics for liquid metals. Phys. Rev. B 47, 558–561 (1993). 10.1103/physrevb.47.558 10004490

[b29] Kresse G. & Joubert D. From ultrasoft pseudopotentials to the projector augmented-wave method. Phys. Rev. B 59, 1758 (1999).

[b30] Dudarev S., Botton G., Savrasov S., Humphreys C. & Sutton A. Electron-energy-loss spectra and the structural stability of nickel oxide: An LSDA + U study. Phys. Rev. B 57, 1505 (1998).

[b31] Rébola A., Klie R., Zapol P. & Öğüt S. First-principles study of the atomic and electronic structures of misfit-layered calcium cobaltite (Ca_2_CoO_3_)(CoO_2_) _1.62_ using rational approximants. Phys. Rev. B 85, 155132 (2012).

[b32] Monkhorst H. J. & Pack J. D. Special points for Brillouin-zone integrations. Phys. Rev. B 13, 5188 (1976).

[b33] Togo A. & Tanaka I. First principles phonon calculations in materials science. Scr. Mater. 108, 1–5 (2015).

