# Supplementary material for: Phononic Structure Engineering: the Realization of Einstein Rattling in Calcium Cobaltate for the Suppression of Thermal Conductivity
Source: Sci Rep. 2016 Jul 26;6:30530. doi: 10.1038/srep30530 (PMC4960590; doi:10.1038/srep30530)
Supplement: Supplementary Information [file srep30530-s1.pdf]

Supplementary Information

Phononic Structure Engineering: the Realization of Einstein Rattling in Calcium Cobaltate for the Suppression of Thermal Conductivity

Ruoming Tian[1], Gordon J Kearley[1,*], Dehong Yu[2], Chris D Ling[3], Anh Pham[1], Jan P Embs[4], Elvis Shoko[5], and Sean Li[1,*]

[1] School of Materials Science and Engineering, UNSW Australia, Sydney, NSW 2052, Australia
[2] Australian Nuclear Science and Technology Organisation, Lucas Heights, NSW 2232, Australia
[3] School of Chemistry, The University of Sydney, Sydney, NSW 2006, Australia
[4] Paul Scherrer Institute, 5232 Villigen PSI, Switzerland
[5] School of Physics, University of the Witwatersrand, Braamfontein 2000, Johannesburg, South Africa
*Authors to whom correspondence should be addressed.

Email: g.kearley@unsw.edu.au; sean.li@unsw.edu.au

Laboratory-based XRD

The XRD measurement was carried out on the pulverized powders of the four synthesised samples by using Panalytical MPD instrument with Cu $K\alpha$ radiation. The results shown in Figure s1 indicate that all the samples can be identified as the $Ca_3Co_4O_9$ single phase by referring to the standard JCPD card.

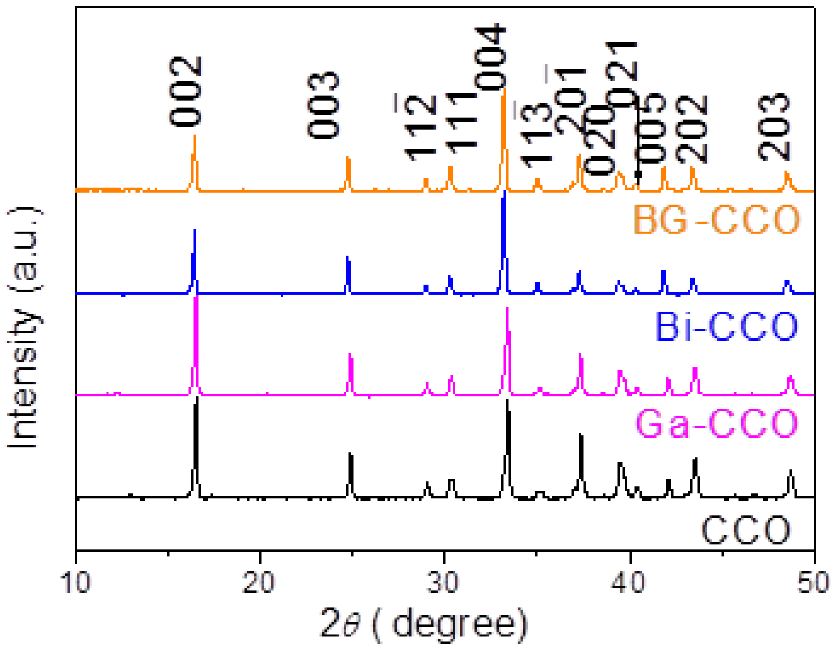

Figure s1 XRD pattern for the undoped sample CCO, Ga singly-doped sample Ga-CCO, Bi singly doped sample Bi-CCO and Bi/Ga co-doped sample BG-CCO.

Synchrotron XRD

Synchrotron XRD was carried out on the undoped and co-doped samples using the Powder Diffraction beamline at the Australian Synchrotron. The data was collected at room temperature under ambient conditions. The energy was set as 13 keV, which was above the Bi *L* edge. The wavelength was 0.82565 (2) Å. Rietveld refinement was carried out using *Jana 2006* program. The initial model and refinement procedures have been discussed in our previous structural studies[1,2] on this material. For the undoped sample, the R factors are R=0.0748 and wR=0.0710 and goodness of fit is 6.99. For the co-doped sample, the R factors are R=0.0911 and wR=0.0704,

and goodness of fit is 3.98. Figure.s2 shows the experimental and refined profiles for the co-doped sample.

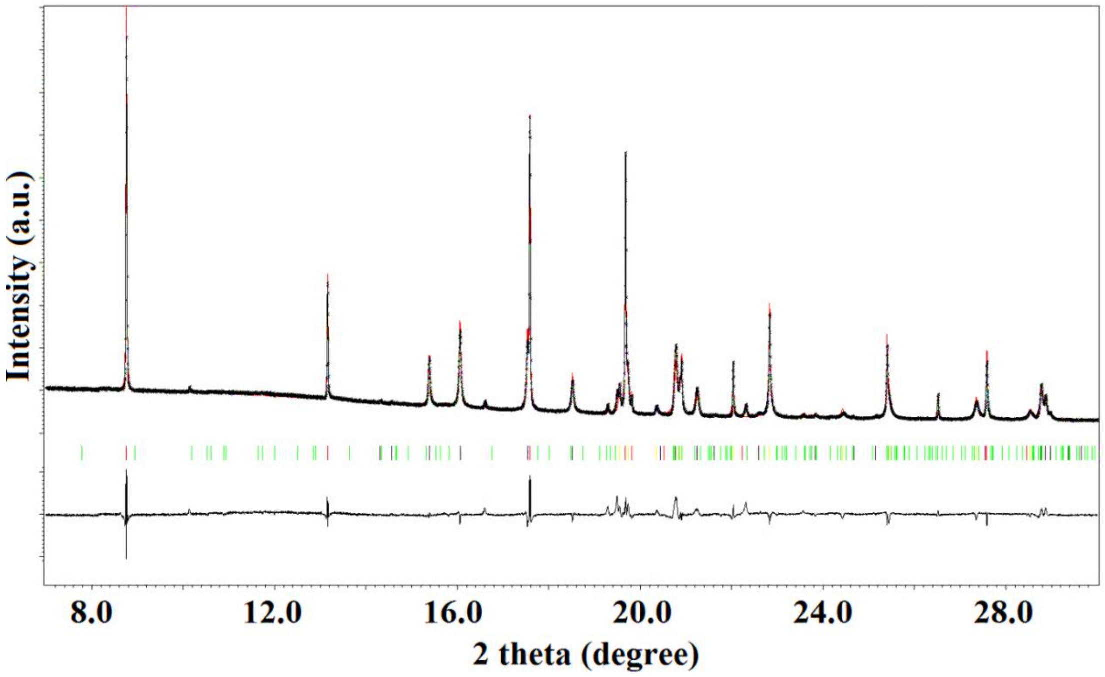

Figure s2. Experimental (dotted line) and refined (red solid line) profile for co-doped sample. The difference between experimental and refined patterns is plotted on the bottom. Overall powder R-factors are R=0.0911 and wR=0.0704.

Electrical Resistivity Measurement

The measurement of electrical resistivity was conducted from 325 to 575K using a ULVAC-ZEM3 system under helium atmosphere slightly above ambient. The error of electrical resistivity was estimated to ~3%, determined by three repeated test for each sample. The result was shown in Table s1, indicating that the resistivity of the Bi and Ga co-doped sample was slightly lower than that of the pristine sample.

Table s1 Measured electrical resistivity for undoped sample (CCO) and Bi/Ga co-doped sample (BG-CCO).

**Temperature (K±5K)**	**325**	**375**	**425**	**475**	**525**	**575**
**CCO (μ Ω m)**	120	121	121	120	116	105
**BG-CCO (μ Ω m)**	107	109	108	106	104	101

References

1 Ling, C. D., Aivazian, K., Schmid, S. & Jensen, P. Structural investigation of oxygen non-stoichiometry and cation doping in misfit-layered thermoelectric $[Ca_2CoO_{3-x}][CoO_2]_\delta$, δ~1.61. *J. Solid State Chem.* **180**, 1446-1455 (2007).

2 Tian, R. *et al.* Ga Substitution and Oxygen Diffusion Kinetics in $Ca_3Co_4O_{9+\delta}$-Based Thermoelectric Oxides. *J. Phys.Chem. C* **117**, 13382-13387, doi:10.1021/jp403592s (2013).
